# Swept-source optical coherence tomography detects anterior-chamber changes in patients with angle-closure after laser peripheral iridotomy

**DOI:** 10.5935/0004-2749.2022-0063

**Published:** 2023-03-20

**Authors:** Bruno L. B. Esporcatte, Norton S. Yanagimori, Guilherme H. Bufarah, Roberto M. Vessani, Luiz Alberto S. Melo Jr, Norma Allemann, Ivan Maynart Tavares

**Affiliations:** 1 Department of Ophthalmology and Visual Sciences, Escola Paulista de Medicina, Hospital São Paulo, Universidade Federal de São Paulo, São Paulo, SP, Brazil

**Keywords:** Gonioscopy, Tomography, Optical coherence, Anterior eye segment, Glaucoma, angle-closure, Iridectomy, Laser therapy, Lasers, Gonioscopia, Tomografia de coerência óptica, Segmento anterior do olho, Glaucoma de ângulo fechado, Iridectomia, Terapia a laser, Lasers

## Abstract

**Purpose:**

This study aimed to compare an teriorchamber parameters acquired by a swept-source anteriorsegment optical coherence tomography before and after laser peripheral iridotomy.

**Methods:**

This study prospectively evaluated 14 patients with primary-angle closure and six patients with primary-angle closure glaucoma. Gonioscopy and anterior-segment optical coherence tomography using the DRI OCT Triton® were performed before and after laser peripheral iridotomy. Anterior-segment optical coherence tomography parameters were studied using scleral spur as reference: angle opening distance at 250, 500, and 750 µm, trabecular-iris space at 500 µm, trabecular-iris angle, trabecular-iris contact length, and iris curvature.

**Results:**

Anterior-segment optical coherence tomography identified 61% of the patients with two or more quadrants closed. Gonioscopy identified more closed angles than anterior-segment optical coherence tomography before laser peripheral iridotomy. In angle parameters, only the angle opening distance of 250 µm at the nasal quadrant was not significantly increased after laser peripheral iridotomy. The iris curvature and trabecular-iris contact length showed a significant reduction induced by the laser procedure. Even in eyes in which gonioscopy did not identify angular widening after laser peripheral iridotomy (n=7), the angle opening distance of 750 µm increased (nasal, 0.15 ± 0.10 mm to 0.27 ± 0.16 mm, p=0.01; temporal, 0.14 ± 0.11 mm to 0.25 ± 0.12 mm, p=0.001) and the iris curvature decreased (nasal, 0.25 ± 0.04 mm vs. 0.11 ± 0.07 mm, p=0.02; temporal, 0.25 ± 0.07 mm vs. 0.14 ± 0.08 mm, p=0.007).

**Conclusions:**

Anterior-chamber changes induced by laser peripheral iridotomy could be quantitatively evaluated and documented by DRI OCT Triton®

## Abstract (pt)

**Objetivo:**

Comparar os parâmetros de câmara anterior obtidos através da tomografia de coerência óptica de segmento anterior antes e após a iridectomia periférica a laser.

**Métodos:**

Quatorze pacientes com fechamento angular primário e seis com glaucoma primário de ângulo fechado foram prospectivamente avaliados neste estudo. Gonioscopia e tomografia de coerência óptica de segmento anterior com DRI OCT Triton® foram realizadas antes e após a iridectomia periférica a laser. Os seguintes parâmetros de tomografia de coerência óptica de segmento anterior, baseados na localização do esporão escleral, foram avaliados: ângulo de abertura angular a 250 µm, 500 µm e 750 µm, área do espaço entre a íris e o trabeculado a 500 µm, ângulo entre a íris e o trabeculado, extensão do contato entre a íris e o trabeculado e curvatura da íris.

**Resultados:**

A tomografia de coerência óptica de segmento anterior identificou 61% dos indivíduos com dois ou mais quadrantes fechados. A gonioscopia identificou mais quadrantes com ângulo fechado do que tomografia de coerência óptica de segmento anterior antes da iridectomia periférica a laser. Quanto aos parâmetros angulares, apenas ângulo de abertura angular a 250 µm no quadrante nasal não aumentou significativamente após a iridectomia periférica a laser. A curvatura da íris e a extensão do contato entre a íris e o trabeculado apresentaram redução significativa induzida pelo procedimento a laser. Mesmo nos olhos em que a gonioscopia não identificou aumento da amplitude angular após iridectomia periférica a laser (n=7), ângulo de abertura angular a 750 µm aumentou (nasal: 0,15 ± 0,10 mm para 0,27 ± 0,16 mm, p=0,01; temporal: 0,14 ± 0,11 mm para 0,25 ± 0,12 mm, p=0,001), e ICURVE diminuiu (nasal: 0,25 ± 0,04 mm vs. 0,11 ± 0,07 mm, p=0,02; temporal: 0,25 ± 0,07 mm vs. 0,14 ± 0,08 mm, p=0,007).

**Conclusões:**

As alterações na câmara anterior induzidas pelo iridectomia periférica a laser puderam ser avaliadas quantitativamente e documentadas pelo DRI OCT Triton®.

## INTRODUCTION

Laser peripheral iridotomy (LPI) communicates the posterior and anterior chambers, equalizing the pressure between the two compartments. This intervention reduces the risk of iris projection toward the cornea, with consequent anterior-chamber angle-closure in eyes with a narrow angle. LPI realization does not guarantee the angle opening in all patients undergoing the procedure, being more effective when a relative pupillary block mechanism causes the angle-closure. In primary-angle closure suspects who underwent LPI, Kumar et al. observed 53.3% persistent iridocorneal contact in at least two quadrants after the procedure. Among these eyes, in 29.2%, the mechanism related to angular closure was plateau iris syndrome^(1)^.

Gonioscopy is the gold standard in evaluating the anterior-chamber angle status. It can identify angle- closure and assess changes after LPI qualitatively^(2)^. However, the clinician’s agreement is poor to moderate^(3,4,5)^, the examination is time-consuming, and patients experience substantial ocular discomfort. Moreover, subjective documentation could affect the evaluation of angular changes after LPI.

Ultrasound biomicroscopy (UBM), Scheimpflug imaging, and anterior-segment optical coherence tomography (AS-OCT) provide a quantitative evaluation of the anterior-chamber anatomy^(6,7,8)^. AS-OCT obtains high-resolution real-time cross-sectional images of anteriorsegment structures. It is widely used for its quick and semi-automatic examination without eye contact and good reproducibility^(9,10)^. DRI OCT Triton® (Topcon Corporation, Tokyo, Japan) is a swept-source OCT designed to obtain images of the retina. The optional module lens attachment provides cross-sectional images of the anterior-chamber, allowing the evaluation of opposed angles in the same axis^(11)^.

Therefore, the use of AS-OCT to monitor the effectiveness of LPI could improve the detection of eyes that did not present angle widening after the laser procedure. This study aimed to evaluate changes in anteriorchamber parameters after LPI using DRI OCT Triton®.

## METHODS

This prospective cross-sectional study was approved by the Institutional Ethics Committee and followed the ethical standards laid down in the Declaration of Helsinki and the International Conference on Harmonization Guidelines for Good Clinical Practice. Written informed consent was obtained from all participants before inclusion in the study.

Consecutive patients referred to the glaucoma division for LPI between March 2017 and May 2018 were enrolled in this study. All participants underwent a comprehensive ophthalmologic examination, including a review of medical history, measurement of best-corrected visual acuity (BCVA), manifest refraction, slit-lamp biomicroscopy, Goldmann applanation tonometry, gonios-copy, and non-dilated fundoscopic examination with a 90D fundus lens (Volk; Mentor, OH, USA). In addition, axial length (AXL) and anterior-chamber depth (ACD) were measured with IOL Master 500® (Carl Zeiss Meditec Inc., Dublin, CA, USA). Participants with a history of ocular trauma or any previous intraocular surgery, including LPI or iridoplasty, were excluded.

### Gonioscopy

Gonioscopy was performed by a glaucoma expert blinded to AS-OCT findings. A glaucoma expert was considered an ophthalmologist who completed at least a 1-year glaucoma fellowship and was attending the institution’s glaucoma service, and five experts participated in this protocol. All participants were evaluated in a darkened room, with a Sussman 4-mirror lens (Ocular Inst., Bellevue, WA, USA) at high magnification (16×), and the eye was maintained in primary gaze position. A 1-mm beam of light was reduced to a narrow slit to evaluate the anterior-chamber angle. Care was taken to avoid directing the beam of light at the pupil. Gonioscopy results were recorded according to the visibility of the anatomical landmarks of the angle (Schwalbe’s line, non-pigmented trabecular meshwork, pigmented trabecular meshwork, scleral spur, and ciliary body) during static gonioscopy. Then, an indentation maneuver was performed, and the presence of iridotrabecular contact signs (imprints or synechiae) by quadrants was recorded.

A quadrant was considered closed on gonioscopy when the pigmented trabecular meshwork could not be identified during static gonioscopy evaluation. Patients were classified as having primary-angle closure (PAC) if two or more quadrants were closed and synechiae or imprints were detected. PAC glaucoma (PACG) was diagnosed based on the combined gonioscopy characteristics of PAC and glaucomatous optic neuropathy (localized or diffuse rim or retinal nerve fiber layer thinning) as assessed by fundoscopic examination.

### AS-OCT

Anterior-segment images were obtained using the swept-source DRI OCT Triton® under dark conditions. All scans were centered on the pupil and acquired on the horizontal axis (3-9 h) to evaluate nasal and temporal quadrants and the vertical axis (12-6 h) to assess superior and inferior quadrants. Inadvertent pressure on the globe was carefully avoided when upper and lower eyelids were displaced to acquire vertical axis images.

**Figure 1:**
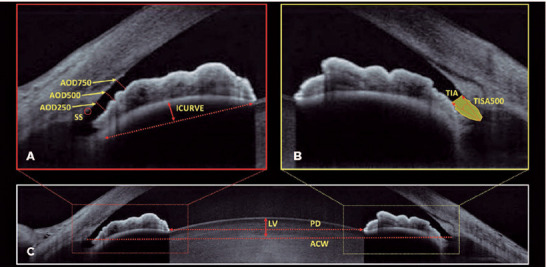
Example images obtained with DRI OCT Triton®. Images A and B show the measurements obtained departing the scleral spur (SS): angle opening distance at 250 µm (AOD250), angle opening distance at 500 µm (AOD500), angle opening distance at 750 µm (AOD750). TIA, trabecular-iris angle; TISA500, trabecular-iris space area at 500 µm; ICURVE, iris curvature. In image C, dashed line represents the anterior-chamber width (ACW), solid line with arrowheads represents the lens vault (LV), and dashed line with arrowheads represents the pupillary distance (PD).

All images were exported to ImageJ® software (V.1.50i) and analyzed by an examiner blinded to gonioscopy results. Only images with clearly discernible scleral spur (SS) and correctly centered on a pupil at vertical and horizontal axes were analyzed. The SS was determined based on the point at which there was a change in the curvature in the corneoscleral aqueous interface, or in the apex of an internal projection of the inner margin of the cornea and trabecular meshwork, and the point at which the interface line between the less reflective ciliary muscle and sclera intersects with the inner corneal margin^(12)^. After manually marking the SS, all quantitative variables were measured (Figure 1). The angle parameters evaluated were the angle opening distance at 250, 500, and 750 µm from the SS (AOD250, AOD500, and AOD750, respectively), trabecular-iris space at 500 µm from the SS (TISA500), trabecular-iris angle (TIA), trabecular-iris contact length (TICL) iris curvature (ICURVE), pupillary distance (PD), and lens vault. The definitions of each parameter were previously described^(13)^. A quadrant was considered closed on AS-OCT if any contact between the iris and corneoscleral surface anterior to the scleral spur could be detected.

### LPI

**Figure 2:**
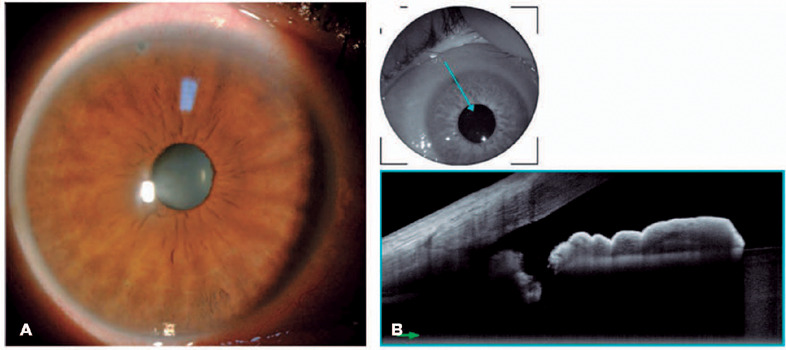
Anterior-segment biomicroscopy highlights the laser peripheral iridotomy (LPI) ostium red arrow in a peripheral crypt at the superior quadrant (A). The patency of the IPL was confrmed with the DRI OCT Triton® (B).

LPI was performed with Nd:YAG-laser (VISULAS YAG III, Carl Zeiss Meditec Inc.) after 15 min of pilocarpine 2% instillation^(14,15)^. Laser was applied at the deepest and most peripheral pseudocrypt, preferably covered by the superior eyelid. Abraham’s lens was used to magnify the area where LPI was created. The power was titrated according to the tissular response starting at 5 mJ. Fluorometholone acetate 0.1% tid and brimonidine tartrate 0.2% bid were prescribed for 1 week. The patency of the ostium was verified by biomicroscopy and AS-OCT 2 weeks after the procedure (Figure 2).

### Statistics

All statistical analyses were performed with Stata® software (Stata version 15; StataCorp, College Station, TX, USA). If both eyes met the inclusion criteria, only the first examined eye was included in this protocol. The means and standard deviations were calculated for continuous variables. The McNemar test was used to compare differences in the distribution of categorical variables. A paired *t*-test and a Wilcoxon signed-rank test were used to compare pre- and post-LPI measurements, depending on the normality test results. The alpha level (type I error) was set at 0.05.

## RESULTS

### Sample description

LPI was indicated in 26 patients after gonioscopy evaluation. Considering all the evaluated quadrants before and after LPI, the SS was not identified in 38 (18%) quadrants. The superior and inferior quadrants presented a lower rate of SS identification (Table 1). Six patients were excluded because of the non-identification of the SS in the nasal or temporal quadrants pre- or post-LPI. Of the 20 patients, 14 were classified as PAC and six as PACG. The mean age of the included patients was 68.3 ± 8.1 (range, 54.2-82.0) years, and 90% were female. The mean BCVA was 0.14 ± 0.19 (range, 0-0.6) logMAR, and the spherical equivalent was +0.64 ± 2.26 D (range, -5.75 to +3.75 D). The mean ACD and AXL were 2.50 ± 0.27 mm (range, 2.12-3.02 mm) and 22.26 ± 0.70 mm (range, 21.03-23.59 mm), respectively. The mean IOP at the baseline visit was 15.2 ± 3.8 (range, 10-22) mmHg. AS-OCT identified 61% of the participants with two or more closed quadrants. Gonioscopy identified more closed angles than AS-OCT in the inferior, nasal, and temporal quadrants before LPI (Table 2).

**Table 1:** Non-identification of scleral spur before and after laser peripheral iridotomy by quadrant

Quadrant	Pre-LPI	Post-LPI	Total	%
Superior	5	10	15	7
Nasal	4	3	7	3
Inferior	6	7	13	6
Temporal	1	2	3	1
Total	16	22	38	1

LPI= laser peripheral iridotomy.

**Table 2:** Gonioscopy and AS-OCT angle-closure detection by quadrants before laser peripheral iridotomy

	Gonioscopy		
	Open angle	Closed angle	p-value
Superior			
AS-OCT			0.06
Open angle	0	5	
Closed angle	0	13	
Nasal			
AS-OCT			<0.001
Open angle	3	13	
Closed angle	0	4	
Inferior			
AS-OCT			0.004
Open angle	0	9	
Closed angle	0	11	
Temporal			
AS-OCT			0.001
Open angle	2	11	
Closed angle	0	7	

AS-OCT= anterior-segment optical coherence tomography.

McNemar test.

### Post-LPI

Two weeks after LPI, gonioscopy identified 7 (35%) eyes remained with two or more quadrants. Considering all the included eyes, angular parameters (AOD250, AOD500, AOD750, TISA500, and TIA) at the nasal and temporal quadrants presented higher values after LPI, although the AOD250 change was not statistically significant at the nasal quadrant. The highest percentual change in angular parameters was observed in AOD750 at the nasal quadrant (74%) and TISA500 (100%) at the temporal quadrant. TICL and ICURVE showed a significant reduction after the laser procedure (Table 3).

**Table 3:** AS-OCT parameter measurements before and after laser peripheral iridotomy

	Pre-LPI	Post-LPI	Difference (Post-Pre)		
Parameters	Mean (SD)	Mean (SD)	Mean (SD)	p-value	% of change
Nasal					
AOD250, _mm_	0.11 (0.08)	0.15 (0.06)	0.03 (0.08)	0.09	36
AOD500, _mm_	0.14 (0.09)	0.22 (0.10)	0.08 (0.09)	<0.001	57
AOD750, _mm_	0.19 (0.11)	0.33 (0.12)	0.14 (0.09)	<0.001	74
TISA500, _mm2_	0.05 (0.04)	0.08 (0.03)	0.03 (0.04)	0.02	60
TIA, _degree_	11.03 (8.92)	18.56 (8.37)	7.53 (7.61)	<0.001	68
TICL, _mm_	0.11 (0.26)	0.03 (0.11)	-0.08 (0.20)	<0.05^#^	-73
ICURVE, _mm_	0.25 (0.05)	0.11 (0.06)	-0.15 (0.08)	<0.001	-56
Temporal					
AOD250, _mm_	0.10 (0.08)	0.14 (0.08)	0.04 (0.06)	0.003	40
AOD500, _mm_	0.14 (0.09)	0.21 (0.12)	0.07 (0.08)	<0.001	50
AOD750, _mm_	0.17 (0.11)	0.30 (0.11)	0.13 (0.09)	<0.001	76
TISA500, _mm2_	0.04 (0.04)	0.08 (0.05)	0.04 (0.04)	<0.001^#^	100
TIA, degree	9.73 (9.91)	15.44 (8.57)	5.71 (8.15)	0.006	59
TICL, _mm_	0.13 (0.23)	0.03 (0.15)	-0.10 (0.17)	0.01	-77
ICURVE, _mm_	0.26 (0.07)	0.12 (0.06)	-0.14 (0.09)	<0.001	-54

AOD250, angle opening distance at 250 m of the scleral spur (SS); AOD500, angle opening distance at 500 m of SS; AOD750, angle opening distance at 750 m of SS; ICURVE, iris curvature; SD, standard deviation; TIA, trabecular-iris angle; TISA500, trabecular-iris space area at 500m of SS. ^#^Wilcoxon signed-rank test. Other comparisons were performed with the paired *t*-test.

**Figure 3:**
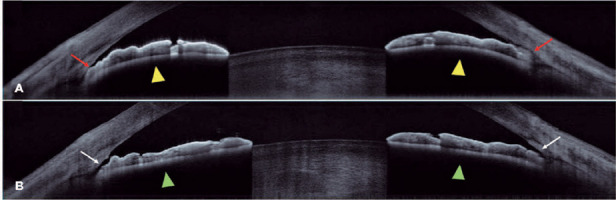
Anterior-chamber section before (A) and after (B) laser peripheral iridotomy (LPI). Before LPI, a narrow angle was detected at the nasal and temporal quadrants (red arrow), and a relative pupillary block was evidenced by iris curvature (yellow arrowhead). After LPI, angle widening was observed (white arrow), and the iris presented a fat contour (green arrowhead).

Lens vault measurement also presented a reduction after LPI (347 ± 171 µm vs. 301 ± 187 µm, p<0.001). Moreover, no significant change was observed in PD (4.23 ± 1.13 mm vs. 4.06 ± 0.93 mm, p=0.24). Figure 3 illustrates the changes in the anterior-chamber induced by LPI.

Although gonioscopy did not identify angular widening after LPI in seven patients, AS-OCT detected changes in the anterior-chamber anatomy. AOD750 varied from 0.15 ± 0.10 mm to 0.27 ± 0.16 mm (p=0.01) and from 0.14 ± 0.11 mm to 0.25 ± 0.12 mm (p=0.001) at the nasal and temporal quadrants, respectively. Likewise, the ICURVE presented a reduction in these patients after LPI at the nasal (0.25 ± 0.04 mm vs. 0.11 ± 0.07 mm, p=0.02) and temporal (0.25 ± 0.07 mm vs. 0.14 ± 0.08 mm, p=0.007) quadrants. All angular parameters sorted by gonioscopy after LPI are summarized in table 4.

**Table 4:** AS-OCT parameter measurements before and after laser peripheral iridotomy sorted by angle status post-procedure

	Open angle post-LPI (n=13)				Angle-closure post-LPI (n=7)			
	Pre	Post	Diff.	p	Pre	Post	Diff.	
Parameter	Mean (SD)	Mean (SD)	Mean (SD)		Mean (SD)	Mean (SD)	Mean (SD)	p
Nasal								
AOD250, _mm_	0.13 (0.09)	0.16 (0.04)	0.03 (0.09)	0.26	0.09 (0.07)	0.12 (0.09)	0.04 (0.06)	0.16
AOD500, _mm_	0.15 (0.09)	0.24 (0.09)	0.09 (0.09)	0.004	0.12 (0.08)	0.19 (0.12)	0.07 (0.09)	0.09
AOD750, _mm_	0.22 (0.11)	0.36 (0.08)	0.14 (0.10)	<0.001	0.15 (0.10)	0.27 (0.16)	0.12 (0.09)	0.01
TISA500, _mm2_	0.06 (0.04)	0.08 (0.02)	0.03 (0.05)	0.11	0.04 (0.03)	0.07 (0.04)	0.02 (0.02)	0.03
TIA, degree	10.93 (8.70)	20.54 (7.37)	9.61 (8.49)	0.004	11.19 (9.95)	15.46 (9.45)	4.26 (4.87)	0.06
TICL, _mm_	0.08 (0.22)	0 (0)	-0.08 (0.22)	0.20	0.17 (0.34)	0.09 (0.19)	-0.08 (0.15)	0.21
ICURVE, _mm_	0.26 (0.06)	0.11 (0.06)	-0.15 (0.09)	0.001	0.25 (0.04)	0.11 (0.07)	-0.14 (0.07)	0.02
Temporal								
AOD250, _mm_	0.09 (0.09)	0.15 (0.08)	0.06 (0.07)	0.005	0.12 (0.07)	0.13 (0.08)	0.01 (0.03)	0.21
AOD500, _mm_	0.14 (0.09)	0.22 (0.11)	0.08 (0.08)	0.005	0.13 (0.08)	0.19 (0.13)	0.06 (0.09)	0.11
AOD750, _mm_	0.18 (0.11)	0.32 (0.10)	0.14 (0.11)	0.001	0.14 (0.11)	0.25 (0.12)	0.12 (0.05)	0.001
TISA500, _mm2_	0.03 (0.04)	0.09 (0.05)	0.05 (0.05)	0.003	0.05 (0.03)	0.07 (0.06)	0.02 (0.03)	0.07
TIA, degree	8.36 (10.99)	15.79 (8.24)	7.43 (8.91)	0.01	12.26 (7.59)	14.77 (9.81)	2.51 (5.75)	0.29
TICL, _mm_	0.15 (0.19)	0 (0)	-0.15 (0.19)	0.016	0.11 (0.30)	0.09 (0.25)	-0.02 (0.05)	0.36
ICURVE, _mm_	0.26 (0.08)	0.10 (0.04)	-0.15 (0.10)	0.001	0.25 (0.07)	0.14 (0.08)	-0.11 (0.06)	0.007

AOD250= angle opening distance at 250 m of the scleral spur (SS); AOD500= angle opening distance at 500 m of SS; AOD750= angle opening distance at 750 m of SS; Diff.= difference; ICURVE= iris curvature; LPI= laser peripheral iridotomy; SD= standard deviation; TIA= trabecular-iris angle; TISA500= trabecular-iris space area at 500 m of SS.

# Wilcoxon signed-rank test. Other comparisons were performed with a paired *t*-test.

## DISCUSSION

The swept-source DRI OCT Triton® is designed for posterior pole analysis and can acquire anterior-segment images with accessory lens modules similar to other OCT devices such as Cirrus® (Carl Zeiss, USA), Spectralis® (Heidelberg Engineering, Germany), and RTvue® (Optovue, USA). This study revealed significant changes in the anterior-chamber anatomy after LPI, detected by DRI OCT Triton®. However, despite the ability of this device to assess longitudinal changes following the laser procedure, the AS-OCT detected fewer closed angles than gonioscopy at the inferior, nasal, and temporal quadrants.

In contrast with previous reports, this study reported a lower rate of angle-closure detection^(16,17,18,19)^. Sakata et al. demonstrated that Visante OCT detected more closed quadrants than gonioscopy in a Singaporean cohort (30% vs. 21%, p<0.001)^(18)^. Moreover, Chong et al. reported that Visante OCT identified more eyes with closed angles than gonioscopy (57.2% vs. 28.1%, p<0.001). Both studies included patients with open angle and angleclosure. As hypotheses for their findings, the authors stated a difference in light intensity emitted to the eye, a possible distortion caused by the gonioscopy lens, and the use of different anatomical landmarks to characterize angle-closure. By contrast, in a North American cohort in which 60% of the patients had PACG, Hu et al. related that gonioscopy detected more closed quadrants than Visante® OCT (48% vs. 18%), similar to our results^(3)^.

The agreement between AS-OCT and gonioscopy varies among devices. When comparing Cirrus® and iVue® OCT, Quek et al. observed a good agreement (AC1=0.72) for angle-closure detection^(5)^. Sakata et al. described a fair agreement between Visante® OCT and gonioscopy in detecting angle-closure in all quadrants (0.40; 95% CI, 0.35-0.45), and this agreement was better at the nasal and temporal quadrants^(18)^.

As expected, in accordance with previous reports^(20,21,22,23,24)^, patients presented an increase in quantitative parameters related to angle opening (AOD250, AOD500, AOD750, TISA500, and TIA) and a decrease in the TICL after LPI. In addition, a significant decrease in ICURVE, related to the resolution of pupillary block, was found by gonioscopy even in a patient who remained to have angle-closure. Thus, some factors may justify changes after LPI in anterior-chamber anatomy in patients with persistent angle-closure by gonioscopic evaluation. First, the angle in these patients could have been inadvertently classified as closed by gonioscopy. Another explanation is related to the image capture over an area of synechial closure; at this point, the iris and angular wall remained adhered after the procedure despite the modifications in the remaining quadrant. Furthermore, our data cannot exclude the possibility of angular closure by various mechanisms, such as the plateau iris syndrome.

To the best of our knowledge, this is the first study using DRI OCT Triton® evaluating anterior-chamber changes after LPI. Recently, Meduri et al. examined the effect of LPI on 18 eyes of 10 patients with Anterion® OCT (Heidelberg Engineering, Germany), a swept-source OCT specifically developed for anterior-segment evaluation. The authors described an average change of +28.26% on AOD750 and +45.5% on TISA500 at the temporal quadrant after LPI^(25)^. In our cohort, the average widening of the nasal and temporal AOD750 was 75%. Moreover, the TISA500 at the temporal quadrant after LPI was 100% higher than the pre-LPI value. Kansara et al. observed a significant change in TISA500 in all quadrants with the CASIA® OCT (Tomey, Japan) in 24 patients with PAC who underwent LPI^(26)^. We observed a significant decrease in lens vault measurement. However, no consensus has been established with this parameter variation, and its changes after LPI probably depend on the angle-closure mechanism^(27,28,29)^.

This study has several limitations. Only one grader evaluated all AS-OCT scans using ImageJ® and manually marked the SS previously for the measurements of angle parameters. The AS-OCT-based grading was not entirely automated, and adjudicating the tissue border landmarks could affect the analysis. Further, we cannot guarantee that AS-OCT images obtained pre- and post-LPI were acquired precisely at the same anatomical position. Finally, the gonioscopic diagnosis was based on the visual inspection of an entire quadrant, whereas the tomographic evaluation, on the analysis of only one point per quadrant, could justify some disagreements between the methods used herein.

In conclusion, this study demonstrates that LPI-induced changes in the anterior-chamber could be quantitatively evaluated and documented by DRI OCT Triton®. Thus, the use of AS-OCT for longitudinal evaluation after laser procedures such as LPI or iridoplasty can provide objective information in contrast to gonioscopy.
